# Domestic

**Published:** 1840-10-10

**Authors:** 


### DOMESTIC.

*Vermont Asylum for the Insane.*—Dr. Rockwell's fourth annual report to the Legislature is characterized, as in past years, by a clear and judicious review of the success of the institution over which he presides. At the close of last year, there were 60 patients; and since, 73 new ones, up to October, were admitted; total enjoying the benefits of the asylum during the year, 143. During the year, 61 were discharged; at the date ot the communication, October 1st, 81 were under his care. All the improvements so judiciously and unhesitatingly introduced within the compass of a few years at Worcester, the McLean Asylum, &c., such as pleasant amusements, religious exercises at regular periods, &c., have been wisely copied by Dr. Rockwell, who is evidently heartily eugaged in the philanthropic duties of the place. The salary is too small, 'tis miserable. The superintendent should at least have enough to encourage him with a hope of having something to retire upon in the winter of his life. This custom of starving medical officers is becoming unpopular. Pay them liberally, and thus secure their undivided services.—*Boston Medical and Surgical Journal.*

*Vermont Academy of Medicine.*—A circular is abroad from this institution, dated March, 1841!—quite in advance. A new faculty has been appointed, or rather new additions have been made to the board, and the prospect of a revivification of the school is encouraging. Prof. Gowdy's death, which took place last year, was a severe loss. Dr. Bryan is placed in the chair of surgery and medical jurisprudence; and Dr. C. L. Mitchell, of New York, is engaged in the new department of pathology. This gentleman is favourably known as the author of a synopsis of auscultation and percussion;—he was a pupil of M. Louis. Six physicians constitute the board of examiners. The lectures will commence on the second Thursday of March next, at Castleton. A winter course of anatomy and physiology, by Dr. Nelson, will begin the first Wednesday of January. He is an accurate teacher, and stands well with those who are judges of what a man should know who professes to instruct others.—*Ibid.*

*Encouragement to Men of Science.*—A committee, consisting of Prof. T. Thomson, Dr. Prout, Prof. Owen, Prof. T. Graham, and Dr. R. D. Thomson, have been appointed in London, with a grant of £200, to make a series of experiments on the *chemistry and physiology of digestion*—and to "*bring over from America Alexis St. Martin,*" upon whom Dr. Beaumont performed his experiments. St. Martin, it will be recollected, was dreadfully wounded at the battle of Plattsburg, during the last war, by having the arch of several ribs and a portion of the fundus of the stomach carried away by a cannon ball. When exhibited in Boston, several years ago, he appeared in excellent health, notwithstanding the aperture into the stomach was frightfully large, so that whatever was swallowed might be seen to drop from the cardiac orifice. Not happening to know where he is now residing, if any correspondents are acquainted with his place of residence, it would be gratifying intelligence to be informed, and it might, perhaps, facilitate the operations of the learned transatlantic commission.—*Ibid.*

